# Approaches for Controlling Illicit Tobacco Trade — Nine Countries and the European Union

**Published:** 2015-05-29

**Authors:** Hana Ross, Muhammad Jami Husain, Deliana Kostova, Xin Xu, Sarah M. Edwards, Frank J. Chaloupka, Indu B. Ahluwalia

**Affiliations:** 1 University of Cape Town, South Africa; 2 Office on Smoking and Health, National Center for Chronic Disease Prevention and Health Promotion, CDC; 3 University of Illinois at Chicago

An estimated 11.6% of the world cigarette market is illicit, representing more than 650 billion cigarettes a year and $40.5 billion in lost revenue (1). Illicit tobacco trade refers to any practice related to distributing, selling, or buying tobacco products that is prohibited by law, including tax evasion (sale of tobacco products without payment of applicable taxes), counterfeiting, disguising the origin of products, and smuggling (2). Illicit trade undermines tobacco prevention and control initiatives by increasing the accessibility and affordability of tobacco products, and reduces government tax revenue streams (2). The World Health Organization (WHO) *Protocol to Eliminate Illicit Trade in Tobacco Products*, signed by 54 countries, provides tools for addressing illicit trade through a package of regulatory and governing principles (2). As of May 2015, only eight countries had ratified or acceded to the illicit trade protocol, with an additional 32 needed for it to become international law (i.e., legally binding) (3). Data from multiple international sources were analyzed to evaluate the 10 most commonly used approaches for addressing illicit trade and to summarize differences in implementation across select countries and the European Union (EU). Although the WHO illicit trade protocol defines shared global standards for addressing illicit trade, countries are guided by their own legal and enforcement frameworks, leading to a diversity of approaches employed across countries. Continued adoption of the methods outlined in the WHO illicit trade protocol might improve the global capacity to reduce illicit trade in tobacco products.

Data on approaches for addressing illicit trade were obtained from a combination of sources from individual countries, including literature searches, reports by international agencies and nongovernmental organizations, industry documents, online data sources by agencies that oversee enforcement, and interviews with in-country experts.* The following 10 most commonly identified approaches were evaluated: 1) licensing, 2) product markers, 3) national recordkeeping, 4) track-and-trace systems, 5) enforcement, 6) export tax, 7) tax harmonization, 8) agreements with tobacco industry, 9) promotion of public awareness, and 10) coordination among agencies. The status of these approaches was assessed in nine countries (Brazil, Canada, Hungary, Italy, Malaysia, Romania, Spain, Turkey, and the United Kingdom [UK]), and EU. These countries were selected based on data availability and participation in the WHO Framework Convention on Tobacco Control (FCTC). EU is described separately from its member states because current approaches used by individual member states may differ from the central EU action plan. Approaches were assessed as of January 2015.

The most common anti-illicit–trade measures were licensing and enforcement (Table 1), which were present in all countries reviewed in this report (Table 2). A total of nine countries employed product markers, most commonly in the form of tax stamps (Table 2). Although requirements for product markers are not included in the centralized EU Tobacco Products Directive, EU member states have incorporated those on an individual basis. Systems for national recordkeeping and agency coordination were established in all countries except Malaysia. Track-and-trace systems, as outlined in the WHO illicit trade protocol, were in effect in Brazil and Turkey, and, in a limited version, in Canada and Hungary; EU and its member states operate a separate system for monitoring the movement of excise goods across their borders. Tax harmonization was employed within EU. Agreements with the tobacco industry were in place in most countries, except for Brazil and Malaysia. Public awareness programs were not widely employed, and export taxes were applied in Brazil and Canada only. While all examined countries were parties to the WHO FCTC, most have not yet ratified or acceded to (i.e., made legally binding) the WHO illicit trade protocol, and only one has thus far acquired accession status (Table 2) (3).

## Discussion

Approaches to address illicit tobacco trade vary across countries. In the sample of countries in this report, the most commonly used approaches included licensing, markers, national recordkeeping, and enforcement, while other measures such as track-and-trace systems and export taxes were not universally employed. Research suggests that the revenue gains from eliminating illicit tobacco trade globally would exceed $31 billion, and might help prevent more than 160,000 tobacco-related deaths per year from 2030 onwards (1). Accordingly, continued adoption of the provisions outlined in the WHO illicit trade protocol and its accession could improve the global capacity to reduce illicit trade in tobacco products and enhance public health.

The WHO illicit trade protocol contains three main elements for addressing illicit trade: 1) controlling the supply chain of tobacco products through track-and-trace systems (Articles 6–13); 2) addressing unlawful conduct and criminal offenses through enforcement means such as seizure and disposal of confiscated products (Articles 14–19); and 3) promoting international cooperation through information sharing, mutual administrative and legal assistance, and extradition (Articles 20–31) (2). The WHO illicit trade protocol emphasizes the importance of national track-and-trace systems, and recommends collection of data on supply-chain movements into a global information sharing database, which would facilitate the coordination of international response (4). Although establishing track-and-trace systems has been identified as a central approach for limiting illicit trade, its implementation is not yet widespread. Some countries may not have the resources to support a fully functioning track-and-trace system, or they may have alternative structures already in place. For example, EU has implemented a substitute computerized system, the Excise Movement and Control System, which differs from the standard track-and-trace model by collecting only limited information in excisable goods, not monitoring duty-paid products, and relaxing the requirement for product markers. Some countries and EU employ agreements with tobacco companies to limit tax evasion, but evidence suggests that the industry-operated monitoring system is subject to limited transparency and insufficient tracing capabilities (5). Turkey is among the countries that have recently implemented track-and-trace systems with noted success; the size of the illicit market has been controlled despite ongoing increases in tobacco taxes in the country (6,7).

The context for illicit tobacco trade globally varies by country. For example, while cross-border smuggling is a primary concern for many countries, the U.S. tobacco market is primarily affected by illicit domestic movement of goods from low-tax to high-tax jurisdictions (8). International experience with tax harmonization across jurisdictions, such as that employed in EU, can provide an example of potential strategies for reducing the size of the domestic illicit market in the United States. Because higher cigarette prices are a primary method for reducing tobacco use (9), an effort to reconcile tax differences across jurisdictions at a shared higher level might help limit tobacco use as well as illicit trade incentives in the United States and other countries.

This report is subject to several limitations. First, it provides a brief summary from a limited number of countries; thus, experiences and approaches from other countries might vary. Second, only the reported presence or absence of an approach was assessed, and differences across countries in the strength of implementation or enforcement were not identified.

Tobacco use is the leading preventable cause of death and disability around the globe, contributing to six million deaths per year (10). Illicit trade in tobacco products undermines global tobacco prevention and control interventions. This report illustrates the diversity of approaches for limiting illicit tobacco trade in a number of countries and EU. These findings underscore the importance of continued adoption of the provisions outlined in the WHO illicit trade protocol to improve the global capacity to reduce illicit trade in tobacco products. Once legally binding (ratified by at least 40 countries), the WHO illicit trade protocol will facilitate international cooperation, a core provision to counteract illicit trade. Further, continued monitoring of the implementation of the WHO illicit trade protocol could counteract the negative economic, societal, and health effects of illicit tobacco trade. Understanding differences across countries in the implementation of the WHO FCTC *Protocol to Eliminate Illicit Trade in Tobacco Products* is important for assessing country-specific needs in implementing this protocol and for identifying best practices that address illicit tobacco trade and reduce tobacco-related disease and death globally.

What is already known on this topic?

Illicit trade in tobacco undermines tobacco control efforts. The WHO Framework Convention on Tobacco Control (FCTC) *Protocol to Eliminate Illicit Trade in Tobacco Products* provides tools for addressing illicit tobacco trade through a package of regulatory and governing principles, and requires FCTC signatories to institute global track-and-trace systems and a global information sharing focal point.

What is added by this report?

There is diversity in the adoption of anti-illicit-trade measures by countries, demonstrating cross-country similarities and differences in main approaches to the standards outlined in the WHO FCTC *Protocol to Eliminate Illicit Trade in Tobacco Products*.

What are the implications for public health practice?

Continued adoption of the methods outlined in the WHO *Protocol to Eliminate Illicit Trade in Tobacco Products* can improve the global capacity to reduce illicit trade in tobacco products and enhance public health. Understanding differences across countries in the status of implementation of the WHO protocol is important for assessing country-specific needs in implementing it, and for identifying best practices in addressing illicit trade.

## Figures and Tables

**TABLE 1:** Definitions of common approaches to address illicit tobacco trade

Approach	Definition
Licensing	Official authorization for engaging in any activity within the tobacco supply chain, from tobacco growing to product manufacturing to product transportation, retail, and export
Markers	Counterfeit-resistant, affixed images on product packaging, most commonly in the form of tax stamps, which indicate date and location of manufacture and the intended retail market
National recordkeeping	Collection of data on the tax liability of tobacco products within country borders or while transiting through individual countries
Track-and-trace	Systems incorporating both markers and national recordkeeping structures to enable tracking of tobacco products throughout the supply chain; tracing the movement of products by transferring tracking data into a global information-sharing database
Enforcement	Commitment to detect and prosecute illicit trade activity
Export tax	Applying a cigarette export tax to reduce the motivation for illegal re-import of exported products
Tax harmonization	Equalizing tax rates across neighboring jurisdictions to lower cigarette price differences across borders
Agreements with industry	Obtaining industry cooperation in improving the security of the supply chain
Public awareness	Disseminating information about the risks associated with illicit tobacco trade; motivating support for enforcement activities
Agency coordination	Coordination between agencies within and across borders to support intelligence gathering, joint customs operations, and sharing of best practices

**TABLE 2:** Implementation of common approaches to address illicit tobacco trade and year of ratification of WHO Framework Convention for Tobacco Control (FCTC) and signing/accession of WHO FCTC *Protocol to Eliminate Illicit Trade in Tobacco Products*, by nine countries and the European Union (EU)

Approach	Brazil	Canada	EU	Hungary	Italy	Malaysia	Romania	Spain	Turkey	UK
Licensing	yes	yes	yes	yes	yes	yes	yes	yes	yes	yes
Markers	yes	yes		yes	yes	yes	yes	yes	yes	yes
National recordkeeping	yes	yes	yes	yes	yes		yes	yes	yes	yes
Track-and-trace	yes	yes		yes					yes	
Enforcement	yes	yes	yes	yes	yes	yes	yes	yes	yes	yes
Export tax	yes	yes								
Tax harmonization			yes	yes	yes		yes	yes		yes
Agreements with industry		yes	yes	yes	yes		yes	yes	yes	yes
Public awareness		yes	yes			yes				yes
Agency coordination	yes	yes	yes	yes	yes		yes	yes	yes	yes
Year ratified WHO FCTC	2005	2004	2005	2004	2008	2005	2006	2005	2004	2004
Year signed/year of accession* WHO illicit trade protocol			2013					2013/2014	2013	2013

**Abbreviations:** UK = United Kingdom; WHO FCTC = World Health Organization Framework Convention for Tobacco Control.

* Accession is an act by which a state signifies its agreement to be legally bound by the terms of a particular treaty.

## Footnotes

* Additional information on sources by country is available at http://tobacconomics.org.
